# New therapies, new challenges: Cystic lung disease after cancer immunotherapy

**DOI:** 10.1016/j.rmcr.2025.102356

**Published:** 2026-01-02

**Authors:** Ananya Venkatesh, David Camacho, Ross Summer, Michael Dong

**Affiliations:** a Department of Internal Medicine, Thomas Jefferson University Hospital, Philadelphia, PA, 19107, USA; b Division of Pulmonary and Critical Care Medicine, Jane and Leonard Korman Respiratory InstituteThomas Jefferson University Hospital, Philadelphia, PA, 19107, USA

**Keywords:** Cystic lung disease, Immunotherapy, Pneumothorax

## Abstract

Immunotherapies have reduced reliance on cytotoxic strategies with their associated high toxicity levels, revolutionizing cancer care. However, these targeted therapies have also increased prevalence of previously rare sequelae, impacting patients experiencing longer survival. We describe a case of endometrial adenocarcinoma with lung metastases, in which tumor resolution following immunotherapy created cystic airspaces, leading to a pneumothorax complication. A 49-year-old female with advanced stage endometrial adenocarcinoma and lung metastasis developed cystic lung lesions in areas of previous metastatic lesions after treatment with Pembrolizumab and Bevacizumab. She developed a pneumothorax secondary to the rupture of one of the large lung cysts, leading to a bronchopleural fistula and required surgical intervention. Structural lung changes, such as cystic lung disease following immunotherapy are emerging complications. As immunotherapy continues to reshape cancer treatment, the emergence of associated complications highlights the need for optimized screening and management strategies for these newly recognized manifestations of cancer therapy.

## Highlights

• Immunotherapy led to cystic lung disease in metastatic endometrial cancer.
• Tumor regression created air-filled cystic cavities, resulting in pneumothorax.
• Bevacizumab and pembrolizumab may increase cystic transformation risk.
• Cystic lung disease is an emerging sequela of modern cancer immunotherapy.

## 1 Introduction

Immunotherapies have reduced reliance on cytotoxic strategies with their associated high toxicity levels, revolutionizing cancer care. However, these targeted therapies have also introduced previously rare sequelae that are increasingly more prevalent and are impacting patients experiencing longer survival with their cancers[1]. Pulmonary malignancies treated with immunotherapy can leave thin-walled cystic cavities. We describe a case of endometrial adenocarcinoma with lung metastases, in which rapid tumor resolution following immunotherapy created pronounced cystic airspaces, leading to a pneumothorax complication.

## 2 Case report

A 49-year-old female with a history of endometrial adenocarcinoma with lung metastasis was admitted with dyspnea and chest pain after a coughing episode.

**Fig. 1:**
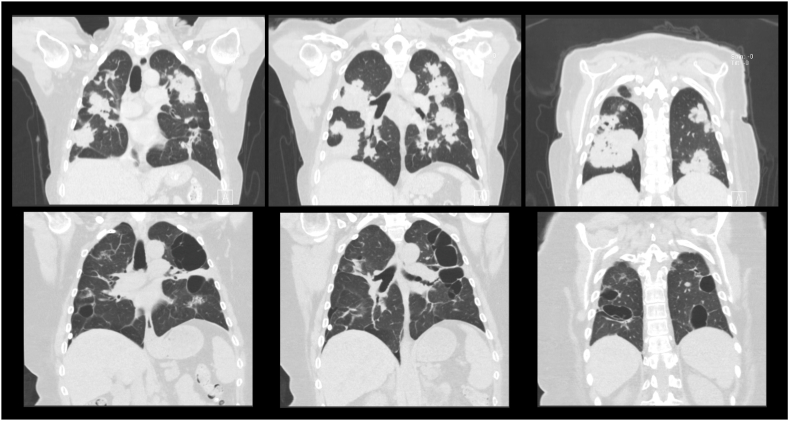
The figure depicts two coronal CT scans. The top row is an initial CT scan demonstrating significant tumor burden, preceding immunotherapy. The bottom row is from a CT scan taken after initiation for monitoring of tumor resorption. Slices are anatomically paired, moving anterior to posterior going left to right. This matching highlights the exact location of metastatic lesions (top row), and the appearance of cystic airspaces only where tumor was previously located (bottom row).

At the time of her initial cancer diagnosis two years prior, she was found to have advanced Stage IVB (cTX, cNX, pM1) endometrial adenocarcinoma with metastasis to bilateral lungs. CT imaging showed numerous confluent pulmonary nodules and masses throughout the lungs constituting approximately 50 % of the lung parenchyma, most notably 9.7 × 5.5cm right lower lobe and 7.4 × 3.5cm left upper lobe pulmonary masses (Fig. 1). She began treatment with systemic chemotherapy with carboplatin and paclitaxel. With disease progression, she then started immunotherapy with pembrolizumab and bevacizumab. A CT scan obtained after this therapy demonstrated extensive thin-walled gas filled cystic lung lesions in areas of previous metastatic lesions (Fig. 1).

On presentation to the hospital, examination was significant for increased work of breathing and decreased breath sounds on the right. A right sided pneumothorax was diagnosed and tube thoracostomy was performed. Her pneumothorax was secondary to the rupture of one of the large lung cysts, leading to a persistent bronchopleural fistula. Despite attempts at conservative management, the fistula persisted. She successfully underwent video-assisted thorascopic surgery (VATS) and surgical pleurodesis. She recovered without complications and was discharged.

## 3 Discussion

While immunotherapies are fundamentally more precise than most chemotherapies, their disruption of common pathways can lead to new and sometimes unexpected sequelae. As survival from advanced stage malignancy improves, these complications will become more prevalent, necessitating the establishment of screening and treatment guidelines.

Among pulmonary complications, anticancer drug induced interstitial lung disease (ILD) is the most commonly reported[2]. However, structural lung changes after therapy are emerging, as seen in our patient. Her rapid tumor regression after immunotherapy resulted in cystic lung disease complicated by pneumothorax, requiring surgical intervention. Pneumothorax associated with pulmonary malignancy treatment is often more difficult to manage, requiring prolonged chest tube drainage, increased surgical intervention, and higher complication risks[3].

Identifying patients at risk for cystic lung disease remains challenging. Tumor size was likely a key predisposing factor in our patient. While mechanistically distinct, if the risk factors are similar to those for patients developing anticancer drug related ILD, then older age, male sex, smoking, previous exposure to chemotherapy, prior pulmonary disease, and poor performance status could increase a patient's risk[4]. Guidelines from the American College of Chest Physicians recommend pulmonary function testing for lung cancer patients to assess pulmonary toxicity risk, but many high-risk patients fall outside these criteria[5]. Though it has not been systematically studied, the type of anticancer drug should have an impact too. In our patient, treatment with Pembrolizumab and Bevacizumab may increase the risk of tumor cavitation and subsequent cystic disease. Pembrolizumab, a PD-1 monoclonal antibody, is primarily associated with dermatologic, gastrointestinal, and hepatobiliary effects, while pulmonary complications like pneumonitis are rare [6]. However, tumor cavitation following PD-1 inhibition is increasingly documented, likely reflecting tumor response rather than treatment toxicity [7]. Bevacizumab, an anti-VEGF monoclonal antibody, has been linked to spontaneous pneumothorax, with anti-VEGF and anti-PD-L1 activity potentially increasing cavitation risk through microenvironment regulation and central necrosis [8,9]. Additional investigation is needed to elucidate the pathophysiology of cystic lung disease in immunotherapy.

The optimal strategies for screening at-risk patients and managing the sequelae and complications of cystic lung disease following cancer treatment remain poorly defined. Expanded inquiry remains essential to understand disease incidence and guide screening for pulmonary complications prior to and during anticancer drug therapy, as well as improve the management of cystic lung disease sequelae. For complications such as complex pneumothoraxes, there should be continued evaluation in outcomes after tube thoracostomy, chemical pleurodesis, surgical pleurodesis, lung volume reduction therapies, and surgical resection [10]. Comprehensive longitudinal care is essential, with consideration for supplemental oxygen therapy, pulmonary rehabilitation, and monitoring for obstructive or restrictive lung disease that may develop.

As immunotherapy continues to reshape cancer treatment, the emergence of cystic lung disease and other associated complications highlights the need for optimized screening and targeted management strategies for these newly recognized manifestations of cancer therapy.

## CRediT authorship contribution statement

**Ananya Venkatesh:** Conceptualization, Resources, Validation, Visualization, Writing – original draft, Writing – review & editing. **David Camacho:** Conceptualization, Resources, Writing – original draft, Writing – review & editing. **Ross Summer:** Resources, Validation, Writing – original draft, Writing – review & editing. **Michael Dong:** Conceptualization, Investigation, Resources, Supervision, Validation, Visualization, Writing – original draft, Writing – review & editing.

## Ethics statement

This report was in accordance with the principles of the Declaration of Helsinki. The patient's family provided verbal and written consent for the publication of the case report.

## Funding

This research did not receive any specific grant from funding agencies in the public, commercial, or not-for-profit sectors.

## Declaration of competing interest

The authors declare that they have no known competing financial interests or personal relationships that could have appeared to influence the work reported in this paper.
